# SOCIAL ISOLATION AMONG MARGINALIZED OLDER ADULTS: APPLYING A UNIFIED MODEL OF RESILIENCE

**DOI:** 10.1093/geroni/igae098.0098

**Published:** 2024-12-31

**Authors:** Andrew Wister

**Affiliations:** Simon Fraser University, Vancouver, British Columbia, Canada

## Abstract

Social isolation is associated with reduced psychological well-being; increased anxiety and depression; compromised physical health; and higher mortality among older adults. Moreover, those who experience various forms of vulnerability and marginalization in society are particularly at risk. We apply the Unified Model of Resilience and Aging (UMRA), an interdisciplinary model that integrates psychological, physiological, and social dimensions of resilience, the latter of which embodies social participation, social isolation, and community belonging. Resilience models, like the UMRA, seek to understand why some individuals, families, and communities recover from adversity better than others, in this case, social isolation and loneliness. The UMRA integrates individual and system-level processes that occur over the life course. According to the UMRA, resilience depends on the activation of internal resources (positive attitude), and activation of external resources (e.g., support from family, friends or organization) which also vary with age and other factors. The UMRA thus includes four domains of environmental resilience situated across the socio-ecological spectrum from individual to global. It also includes four system-level (organizational) functions used by the National Academy of Sciences Resilience Model: 1) Planning for adverse events requires reductions in risk in response to an identified threat. 2) Absorption of stressors and outcomes associated with an adversity is necessary to initiate resilience through recovery and adaptation. 3) Recovery occurs through various forms of short and long-term strength-based resilience. 4) Adaptation relates to changes in the system to promote future resilience. We apply the UMRA to frame causes and consequences of social isolation.

